# Changes in body awareness, body appreciation, and self-esteem during a university aerobics course: a quasi-experimental study

**DOI:** 10.3389/fpsyg.2026.1952535

**Published:** 2026-10-19

**Authors:** Sihua Liu, Yuanyuan Ma

**Affiliations:** 1 Nanchang Institute of Technology, Nanchang, Jiangxi, China; 2 Henan Finance University, Zhengzhou, Henan, China

**Keywords:** aerobics, body appreciation, body awareness, cluster-robust inference, quasi-experimental design, self-esteem, undergraduate students

## Abstract

Undergraduate students have a higher risk of negative body image and low self-esteem, and the majority of exercise interventions in this population focus on fitness or weight rather than how students see their bodies. This quasi-experimental study examined whether a 12-week university aerobics course was associated with greater week-12 body awareness, body appreciation, and self-esteem than a comparison physical-education course, while controlling for baseline scores, and whether early change in body awareness and subsequent change in body appreciation statistically accounted for change in self-esteem. Two hundred forty undergraduates from a single university in China, nested within eight intact physical-education classes (four aerobics, four comparison), were assessed at baseline and at weeks 4, 8, and 12. Because the condition was assigned at the class level, the class, rather than the individual student, served as the unit of condition assignment and the independent replication. The primary week-12 analysis used baseline-adjusted ANCOVA, with class-level clustering taken into account in the inference. At week 12, the aerobics group had higher adjusted scores than the comparison group in terms of body awareness [*b* = 0.43, 95% CI (0.29, 0.56)], body appreciation [*b* = 0.18, 95% CI (0.09, 0.28)], and self-esteem [*b* = 0.77, 95% CI (0.62, 0.91)]. In an exploratory class-cluster-bootstrapped mediation model, the indirect estimate following the hypothesized sequence from course condition through change in body awareness and body appreciation to change in self-esteem was different from zero [0.146, 95% CI (0.079, 0.240)]; however, this estimate was similar in magnitude to the two individual indirect pathways and should be considered descriptive and hypothesis-generating rather than evidence for a uniquely supported sequential mechanism. Students in the aerobics course scored higher on body awareness, body appreciation, and self-esteem at week 12 than students in a comparison physical-education course, and exploratory analyses were consistent with body awareness and body appreciation accounting for part of this association. Because the assignment was non-random, only eight intact classes were included, and the comparison condition was not fully described, the findings should be interpreted as between-course differences rather than evidence of an aerobics-specific causal effect.

## Introduction

Body image and self-esteem are consistently studied as indicators of psychological well-being among emerging adults, and university life is a period of increased susceptibility to difficulties in both domains. Leaving home, experiencing new social-comparison contexts, and internalizing appearance-related norms circulating on campus and online contribute to an increased risk of negative body evaluation and diminished self-worth among undergraduate students (Vartanian and Dey, 2013). Exercise interventions aimed at improving how students feel about their bodies have often emphasized weight management, fitness gains, or appearance change—outcomes that can themselves reinforce an appearance-focused relationship with the body rather than improve body image. An alternative perspective argues that body image may improve more sustainably when interventions strengthen how a person attends to and experiences the body from the inside, rather than how the body looks from the outside (Alleva and Tylka, 2021; Menzel and Levine, 2011; Piran, 2017). In the present study, body awareness refers to self-reported habitual attentiveness to perceived changes in internal bodily states, as operationalized by the Body Awareness Questionnaire (Shields et al., 1989). This construct should be distinguished from objective interoceptive accuracy, which concerns performance in detecting internal physiological signals. Thus, a higher body-awareness score in this study indicates greater self-reported attentiveness or sensitivity to bodily changes, not necessarily greater objective accuracy in perceiving internal bodily signals (Mehling et al., 2009). Recent evidence continues to support exercise as a correlate of more favorable body-related self-evaluation among undergraduate and general adult samples (Matheson et al., 2025; Shi et al., 2024; Zhang et al., 2024).

University aerobics courses offer a distinctive context for examining this embodiment-based account. Aerobics instruction typically involves sustained, rhythmic, whole-body movement, and may draw attention to breathing, posture, coordination, and the internal sensations of exertion and recovery. However, the extent to which any particular course emphasizes these elements depends on how it is taught (described further under the Intervention section, below), and generic choreographed aerobics does not, unlike yoga, typically include explicit interoceptive or mindfulness instruction as part of its standard format. These features plausibly relate to body awareness as defined above, namely self-reported habitual attentiveness to internal bodily changes (Shields et al., 1989; Mehling et al., 2009). If sustained engagement with rhythm, breath, and bodily feedback over a semester of aerobics practice cultivates greater self-reported body awareness, this heightened awareness is theorized, in turn, to redirect self-evaluation away from appearance and toward a more accepting, functionality-oriented relationship with the body (Daubenmier, 2005; Impett et al., 2006). This theorized second step draws on evidence collected in yoga practitioners specifically, a movement modality with more explicit interoceptive and mindfulness instruction than typical aerobics classes; its extension to aerobics here is a theoretical inference rather than a direct empirical finding, and is treated as such throughout. Body appreciation is consistently associated with broader self-esteem in the body-image literature (Tylka and Wood-Barcalow, 2015a, 2015b) and may represent one psychological pathway through which body-focused movement relates to global self-worth, motivating the exploratory statistical pathway examined here: course condition (aerobics vs. comparison) → body awareness → body appreciation → self-esteem.

Three theoretical strands motivate the present hypotheses. First, the construct of body awareness, as operationalized by the Body Awareness Questionnaire (Shields et al., 1989), captures self-reported habitual attentiveness to internal bodily processes; structured aerobics instruction, to the extent that it draws attention to breath, rhythm, and internal exertion cues, is theoretically well suited to strengthening this attentiveness over time. Second, embodiment perspectives on body image (Menzel and Levine, 2011; Piran, 2017) propose that physical activities emphasizing internal experience shift the basis of self-evaluation from how the body looks to how it feels and functions, a redirection associated with more positive, less self-objectified body regard in body-attentive movement practices such as yoga (Daubenmier, 2005). Third, functionality-based models of positive body image (Alleva and Tylka, 2021; Tylka and Wood-Barcalow, 2015a) treat body appreciation as consistently associated with, and may represent one psychological pathway toward, broader self-esteem. Taken together, these frameworks motivate testing body awareness and body appreciation as sequential, rather than only parallel, correlates of change in self-esteem.

The present study examined whether students enrolled in a 12-week aerobics course showed higher week-12 body awareness, body appreciation, and self-esteem than students enrolled in a comparison physical-education course, adjusting for baseline levels and prespecified covariates. It further explored, on an exploratory basis, whether change in body awareness and change in body appreciation statistically accounted for change in self-esteem. The following hypotheses were tested. H1–H3 are the study’s primary hypotheses based on the theoretical framework above; H4–H5 are secondary association hypotheses; and H6–H7 are exploratory mechanistic hypotheses. None of these hypotheses were formally pre-registered with a dated, timestamped analysis plan, so “primary” here reflects the designated analytic priority within this study rather than documented ex-ante commitment.

H1. Relative to the comparison group, the aerobics group will show significantly higher body awareness at week 12 after adjustment for baseline body awareness and prespecified covariates.

H2. Relative to the comparison group, the aerobics group will show significantly higher body appreciation at week 12 after adjustment for baseline body appreciation and prespecified covariates.

H3. Relative to the comparison group, the aerobics group will show significantly higher self-esteem at week 12 after adjustment for baseline self-esteem and prespecified covariates.

H4. Body awareness will be positively associated with body appreciation.

H5. Body appreciation will be positively associated with self-esteem.

H6 (exploratory). Body awareness will statistically mediate the association between course condition (aerobics vs. comparison) and body appreciation.

H7 (exploratory). Body awareness and body appreciation will together statistically mediate the association between course condition and self-esteem via a sequential (chain) indirect pathway.

## Materials and methods

### Study design

This study used a two-condition, four-wave quasi-experimental design embedded within existing university physical-education courses. Eight intact classes participated, comprising four aerobics classes and four comparison physical-education classes; each class enrolled more students than were sampled for this study, and the analytic participants were selected within each class as described under Participants below. Students selected their courses before recruitment into the study; therefore, the research team did not randomly assign individual participants or classes, although it randomly selected participants within each class for inclusion (see Participants section). Assessments were conducted at baseline (week 0) and at weeks 4, 8, and 12. Body awareness, body appreciation, and self-esteem were measured at every wave; the exploratory mediation model (described under the Statistical Analysis section, below) uses week-4 body awareness, week-8 body appreciation, and week-12 self-esteem as temporally ordered measures consistent with the hypothesized sequence. However, because the underlying change intervals overlap in time (e.g., change in body appreciation from T0 to T2 partly overlaps the interval over which body awareness also changed), this design does not establish strict temporal precedence among the three constructs (see Limitations section).

### Participants

Participants were recruited from Henan Finance University, Henan Province, China. Each of the eight participating classes enrolled more students than were included in this study. Eligibility (described below) was assessed first using each class’s full roster, after which a simple random sample of 30 eligible students was selected from each class’s eligible pool. The exact number of students originally enrolled in each class, the number who did not meet eligibility criteria, and whether any student who was sampled declined or was replaced are not reported in full here (see Limitations section). This yielded 240 undergraduate students in total (149 female, 88 male, 3 non-binary; mean age 19.94–20.04 years across conditions): 120 in the aerobics condition (4 classes, 30 randomly sampled students per class) and 120 in the comparison condition (4 classes, 30 randomly sampled students per class). Because condition was determined by which class a student’s course enrollment placed them in, and only within-class sampling—not the assignment of classes to condition—was randomized, the design remains a class-level quasi-experiment rather than a randomized controlled trial. Accordingly, the class, rather than the individual student, was the effective unit of condition assignment and independent replication for between-condition inference. The target of 30 participants per class was a pre-specified pragmatic sampling quota intended to provide equal representation from each of the eight intact classes while keeping repeated data collection across four assessment waves manageable. It was not derived from an *a priori* statistical power calculation. Because conditions varied at the class level, inferential precision for between-condition comparisons depended primarily on the eight classes (four per condition), rather than on the total number of 240 students. Accordingly, the design has limited ability to detect modest class-level differences or to estimate between-class variability precisely.

Eligible students were regularly enrolled undergraduates aged 18–24 who were medically cleared for moderate-intensity exercise and provided informed consent; eligibility was assessed before the random sampling step described above. Students with a medical condition contraindicating moderate-intensity exercise or concurrent enrollment in competitive dance or aerobics training were excluded from the eligible pool before sampling. A binary indicator of any prior aerobics or dance experience (present in 20.8% of the aerobics group and 25.0% of the comparison group; Table 1) was recorded but does not by itself indicate formal, systematic aerobics instruction; this distinction is noted because the two are related but not equivalent.

**Table 1:** Baseline sample characteristics by condition.

Characteristic	Aerobics (*n* = 120)	Comparison (*n* = 120)	SMD	Test, *p*
Age, years, M (SD)	19.94 (1.12)	20.04 (1.26)	−0.08	*p* = 0.515
BMI, kg/m^2^, M (SD)	22.67 (2.79)	22.57 (3.09)	0.03	*p* = 0.789
Sex, female/male/non-binary, *n*	72/46/2	77/42/1	—	*p* = .693^a^
Year of study, 1/2/3/4, *n*	34/25/39/22	30/31/30/29	—	*p* = 0.387
Prior aerobics experience, *n* (%)	25 (20.8%)	30 (25.0%)	—	*p* = 0.539
Baseline physical activity, MET-min/week, M (SD)	1576.0 (586.4)	1550.3 (625.7)	0.04	*p* = 0.743
Baseline body awareness (1–7), M (SD)	4.02 (0.69)	4.07 (0.70)	−0.07	*p* = 0.597
Baseline body appreciation (1–5), M (SD)	3.28 (0.66)	3.23 (0.70)	0.08	*p* = 0.557
Baseline self-esteem (10–40), M (SD)	27.82 (5.19)	28.39 (5.09)	−0.11	*p* = 0.391

a Monte Carlo exact test (20,000 permutations); all other categorical comparisons used *χ*^2^.

SMD, standardized mean difference (Cohen’s *d* on baseline values).

Follow-up questionnaires were administered during scheduled course sessions at weeks 4, 8, and 12. Students who were absent on the initial assessment day were offered one make-up assessment within 3 days, which contributed to the complete four-wave follow-up rate reported below. The analytic sample consisted of 240 participants with complete data across all four assessment waves. The analytic dataset contained a variable labeled as the number of sessions attended; however, the original attendance registers and the operational definition of attendance were not available, so this variable is not reported as a reliable index of intervention exposure (see Limitations section).

Figure 1 provides a CONSORT-style flow diagram adapted for the quasi-experimental design to summarize class participation, eligibility assessment, within-class sampling, and follow-up.

**Figure 1:**
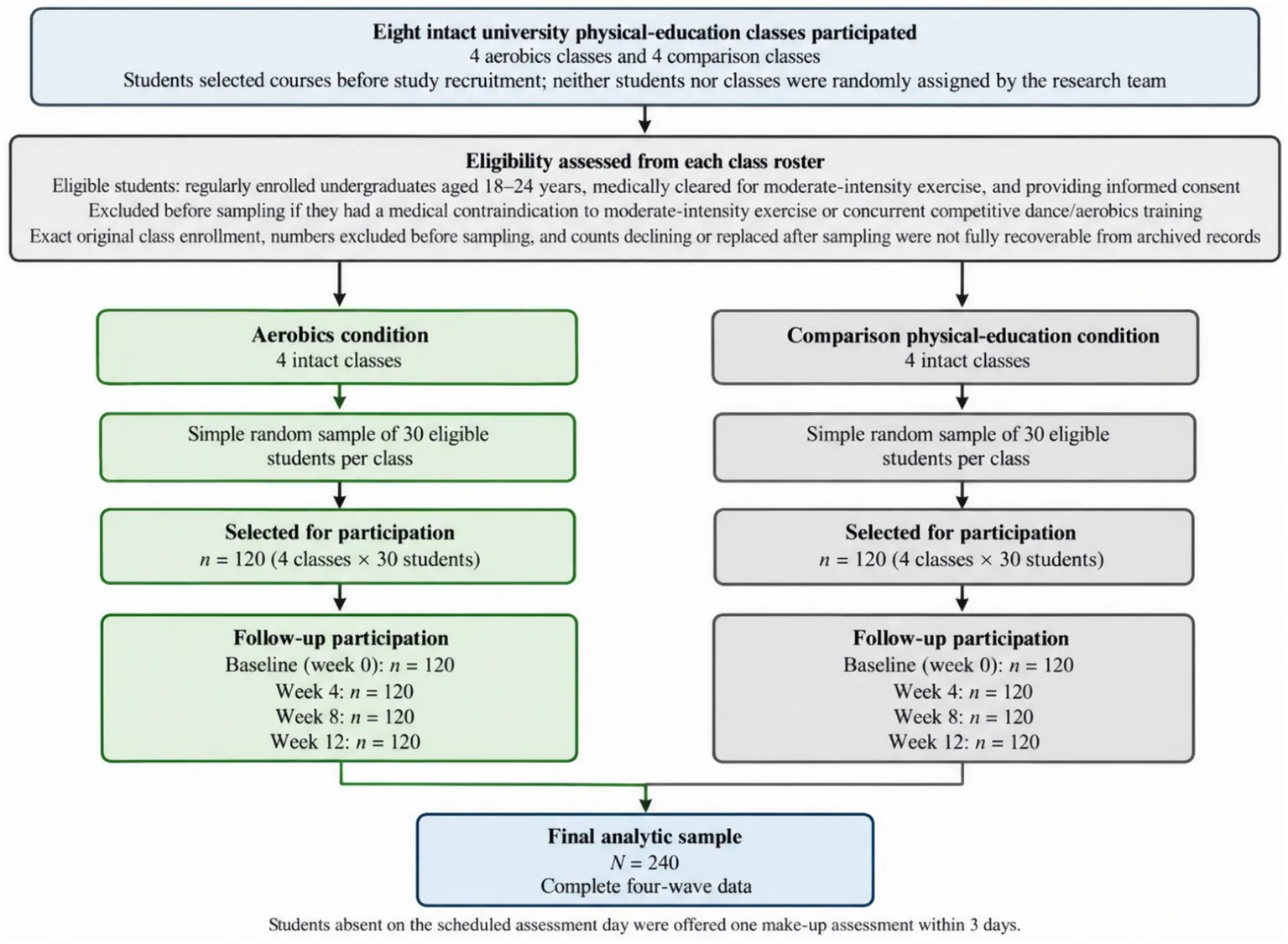
Participant flow diagram adapted for the quasi-experimental design. Exact enrollment, exclusion, and post-sampling decline/replacement counts were not fully recoverable from archived records.

### Intervention

Intervention and comparison condition. The aerobics course was scheduled once weekly for 12 consecutive weeks, with each session lasting 60 min. The archived study records did not provide a sufficiently detailed session-by-session description to reconstruct specific session components, week-to-week progression, instructor qualifications, or prescribed exercise intensity of the aerobics course. All four aerobics classes were taught by the same instructor. The available records also did not permit verification that interoceptive awareness, mindfulness, or explicit body-awareness instruction was formally taught as part of the aerobics course. The comparison condition comprised four existing usual-practice university physical-education classes, reported in the analytic documentation as being scheduled at the same frequency and duration. The archived study records did not identify the specific activities delivered in the comparison classes or provide information on their week-by-week curriculum, session structure, instructor assignment, instructor qualifications, use of music or synchronized movement, attendance-recording procedures, or exercise-intensity monitoring. The available records also did not contain direct measures of instructor enthusiasm, perceived novelty or enjoyment, social interaction or group cohesion, or participant expectations regarding the courses. Exercise intensity and implementation fidelity were not objectively evaluated in either condition. The comparison condition is therefore treated throughout as an incompletely characterized usual-practice comparator rather than as a standardized active control.

### Measures

Body awareness was assessed using a Chinese-language version of the Body Awareness Questionnaire (BAQ; Shields et al., 1989), an 18-item instrument scored as an item mean on a 1–7 response format, with higher scores indicating greater self-reported habitual attentiveness to internal bodily processes; the BAQ is a self-report measure of perceived bodily sensitivity and does not assess objective interoceptive accuracy. Body appreciation was assessed with the Body Appreciation Scale-2 (BAS-2; Tylka and Wood-Barcalow, 2015b), a 10-item measure scored as an item mean (1–5). Self-esteem was assessed with the Rosenberg Self-Esteem Scale (RSES; Rosenberg, 1965), a 10-item measure (5 positively and 5 negatively worded items; the 5 negatively worded items were reverse-coded according to the prescribed scoring rule before the 10 items were summed) scored as a summed total (10–40).

Baseline physical activity was assessed using the Chinese version of the International Physical Activity Questionnaire–Short Form (IPAQ-SF; Macfarlane et al., 2007). Following the standard IPAQ scoring protocol, walking, moderate-intensity activity, and vigorous-intensity activity were assigned MET values of 3.3, 4.0, and 8.0, respectively. For each activity category, MET-min/week was calculated as MET value × minutes per day × days per week, and the category-specific values were summed to obtain total weekly physical activity. Questionnaire data were collected electronically using Wenjuanxing. Item responses were exported from Wenjuanxing, and the final composite scores used for analysis were calculated in SPSS. For the BAQ, the composite score was calculated as the mean of the 18 items on the 1–7 response scale. For the BAS-2, the composite score was calculated as the mean of the 10 items on the 1–5 response scale. For the RSES, the five negatively worded items were reverse-coded according to the prescribed scoring rule before summing the 10 items to obtain a total score ranging from 10 to 40.

During data processing, designated class representatives checked scoring for their respective classes in SPSS, including applying the reverse-coding rule for the negatively worded RSES items. The scoring files and procedures were cross-checked across classes, and range checks confirmed that all retained composite scores fell within the theoretical ranges of the respective instruments.

During preparation of the de-identified analytic dataset, only the resulting composite scale scores were retained; the original item-level responses were not preserved. Consequently, the present archived dataset does not permit independent reconstruction of the composite scores from individual items. Sample-specific internal consistency (Cronbach’s *α* or McDonald’s *ω*) cannot be calculated, measurement invariance across time or condition cannot be tested, and the original item-level application of the RSES reverse-scoring procedure cannot now be independently re-verified. These constitute substantive measurement limitations of the present study.

### Statistical analysis

Analyses were conducted in Python 3.12.3 with pandas 3.0.2, statsmodels 0.14.6, SciPy 1.17.1, and pingouin 0.6.1. The analytic file contained no missing values (see Participants section, above). Descriptive statistics and standardized mean differences (Cohen’s *d* computed on baseline values) were used to characterize baseline group differences, alongside independent-samples *t*-tests (continuous variables), chi-square tests (categorical variables with all expected cell counts ≥5), and a Monte Carlo exact test (20,000 permutations) for the sex comparison, whose contingency table included one small expected cell.

Although 240 students contributed outcome data, between-condition inference was based on only eight independently observed classes (four per condition); the student-level sample size therefore should not be interpreted as 240 independent experimental units. The primary analysis for H1–H3 was a baseline-adjusted ANCOVA at week 12 (T3): each outcome’s T3 score was regressed on condition, its own T0 score, sex, age, BMI, baseline physical activity, and prior aerobics experience, with cluster-robust (CR1) standard errors clustered on class. These covariates were selected *a priori* because sex, age, BMI, baseline physical activity, and prior aerobics experience were plausible baseline correlates of both course selection and study outcomes; baseline physical activity and prior aerobics experience also captured pre-existing exercise exposure. To facilitate comparison across outcomes measured on different scales, a standardized coefficient for the condition effect was additionally reported for each primary week-12 model. The binary condition indicator was retained in its original 0/1 coding, and the outcome was standardized so that the coefficient represents the adjusted between-condition difference in standard-deviation units. Because only eight clusters were available, this cluster-robust inference was corroborated with an exhaustively enumerated wild cluster bootstrap-t procedure under the null of no condition effect, using Rademacher weights (Cameron et al., 2008). With 8 clusters, there are exactly 2^8^ = 256 possible sign-weight combinations; all 256 were enumerated exhaustively rather than approximated by random resampling. Here, “exhaustive” refers only to enumeration of all possible Rademacher sign combinations for the eight observed classes; it does not imply randomization-based exact inference, because classes were not randomly assigned to conditions. At each of the 256 combinations, the model was refit on the sign-perturbed data and its own cluster-robust *t*-statistic recomputed (rather than comparing raw coefficients), and the reported *p*-value is the two-sided proportion of the 256 bootstrap *t*-statistics at least as extreme as the observed one—the studentized (“wild bootstrap-t”) refinement that Cameron et al. (2008) show improves on comparing raw bootstrap coefficients. Because Rademacher sign combinations pair up (a combination and its complete sign reversal produce bootstrap t-statistics equal in absolute value), every attainable |t*| value occurs an even number of times among the 256 combinations, so the two-sided wild-bootstrap-t *p*-value based on exhaustive enumeration of all 256 sign combinations is bounded below by 2/256 ≈ 0.0078, not 1/256 (see Results section, below). The wild bootstrap-t procedure is used here to obtain this exact *p*-value only; because the sign-flip distribution is constructed under the imposed null of no condition effect, it is not centered on the actual point estimate, and percentiling it directly would not yield a valid confidence interval for the coefficient. The cluster-robust (CR1) interval reported alongside each estimate is therefore used as the confidence interval throughout; a separate wild-bootstrap confidence interval is not reported. Holm’s sequential correction was applied across the three primary week-12 outcome tests (body awareness, body appreciation, self-esteem), using the wild-bootstrap-t *p*-values as input; both raw and Holm-adjusted *p*-values are reported. Week-4 and week-8 between-condition contrasts were analyzed with the same baseline-adjusted, cluster-robust specification (without the wild bootstrap or Holm correction) and were treated as secondary, descriptive trajectory analyses rather than additional primary tests. As a sensitivity analysis, the primary week-12 contrasts were also estimated on raw T0-to-T3 change scores (without separately entering T0 as a covariate); this produced substantively similar conclusions and is not reported in full.

Effect sizes are reported as Hedges’ *g* (bias-corrected standardized mean difference in T0-to-T3 change scores, with a small-sample correction), calculated descriptively from individual change scores; because *g*’s conventional formula treats participants as independent, its 95% confidence interval was instead estimated by a class-cluster bootstrap (5,000 resamples of intact classes, described below), so that the reported interval—unlike the point estimate—reflects the class-level unit of assignment.

As an omnibus, descriptive check on the overall shape of each outcome’s four-wave trajectory, a linear mixed-effects model (fixed effects for condition, categorical time, and their interaction; random intercept for participant) was compared, via a likelihood-ratio test, against a reduced model omitting the interaction term; both models in each comparison were fitted by maximum likelihood (not restricted maximum likelihood) because likelihood-ratio tests between models with different fixed-effects structures require ML estimation. This omnibus model does not incorporate class-level clustering or covariates and is reported as descriptive context for the primary week-12 ANCOVA results above, not as an independent primary test.

As a class-level sensitivity analysis, mean T0-to-T3 change was also computed for each of the eight classes, and the four aerobics-class means were compared with the four comparison-class means using the class (not the participant) as the unit of analysis; this analysis has very limited statistical power given only four clusters per condition but does not rely on treating individual students as independent observations. No observed-effect *post hoc* power calculation was performed, because such calculations largely restate the obtained test result. As an illustrative sensitivity benchmark, a simple two-sided comparison based on four independent classes per condition would require a very large standardized class-level difference (approximately *d* = 2.4) to achieve 80% power at *α* = 0.05 under conventional independent-class *t*-test assumptions. This value illustrates the severe information constraint imposed by having four classes per condition; it is not an exact minimum detectable effect for the covariate-adjusted cluster-robust ANCOVA and is not directly comparable with the participant-level Hedges’ *g* values reported below.

H4 and H5 concerned the longitudinal association between the focal constructs, but because both constructs are also associated with condition (see Results section, below), condition was included as a covariate to reduce confounding of the focal association by shared intervention exposure, alongside the corresponding baseline scores (week-8 body appreciation regressed on week-4 body awareness, baseline body awareness, baseline body appreciation, and condition for H4; week-12 self-esteem regressed on week-8 body appreciation, baseline body appreciation, baseline self-esteem, and condition for H5); these models did not include the fuller covariate set (sex, age, BMI, physical activity, prior experience) used in the primary intervention models. Both regressions used cluster-robust (CR1) standard errors clustered on class rather than treating participants as independent. Zero-order correlations are also reported for descriptive comparison.

The exploratory sequential mediation hypotheses (H6, H7) were tested on baseline-to-follow-up change scores (Δ body awareness = T1 − T0; Δ body appreciation = T2 − T0; Δ self-esteem = T3 − T0), matching the temporal structure described in the Study Design section. An identical covariate set (condition, sex, age, BMI, baseline physical activity, prior aerobics experience) was entered in every equation of the path model, so that a coherent decomposition of the total effect into direct and indirect components could be obtained; an earlier check that instead conditioned each equation only on its own outcome’s baseline, without a shared covariate set, produced unstable estimates because of substantial multicollinearity between same-construct baseline and follow-up scores, and is not used here. The path model specified condition (X) predicting Δ body awareness (M1), which together with X predicted Δ body appreciation (M2), which together with X and M1 predicted Δ self-esteem (Y), following the regression-based multiple-mediator framework of Preacher and Hayes (2008); see also Hayes (2018). Confidence intervals were estimated by resampling intact classes (5,000 iterations; at each iteration, four aerobics classes and four comparison classes were drawn with replacement, retaining all students in each resampled class, and ordinary least-squares point estimates were recomputed on the pooled resampled data), which is the resampling unit that matches how condition was actually assigned; because each bootstrap iteration re-estimates point estimates on pooled data rather than re-computing cluster-robust standard errors keyed to the original class labels, repeated selection of the same class within an iteration does not require relabeling it as a distinct pseudo-cluster. Given the quasi-experimental design, the small number of classes, and the overlapping change intervals noted in Study Design, the mediation results were interpreted as exploratory, descriptive evidence consistent with, rather than confirmation of, the proposed mechanism. Statistical significance for all tests was set at *α* = 0.05, two-tailed.

## Results

### Baseline characteristics and class-level dependence

Baseline characteristics were broadly similar across conditions (Table 1), although the quasi-experimental design does not rule out imbalance in unmeasured characteristics. Standardized mean differences (SMD) for all continuous baseline variables were small (|SMD| ≤ 0.11), and the sex distribution did not differ significantly between conditions (Monte Carlo exact *p* = 0.693, used because one cell—non-binary participants—had an expected count below 5).

Unconditional (intercept-only) mixed models estimated small baseline intraclass correlations attributable to class membership (ICC < 0.001 for body awareness and self-esteem; ICC = 0.03 for body appreciation). A small baseline ICC indicates that the eight classes did not differ much from one another before the intervention, but it does not by itself establish that class-level dependence was negligible during the intervention period: condition was assigned at the class level, so any class-specific instructor effects, scheduling effects, or group dynamics that emerged over the 12 weeks could induce dependence among students in the same class regardless of the baseline ICC. Because only eight classes were available, these data alone could not estimate class-level dependence with high precision. All primary between-condition contrasts therefore used cluster-robust standard errors and were accompanied by a wild cluster bootstrap and a class-level (unit-of-analysis) sensitivity check, described in the Results section, below.

### Change in body awareness, body appreciation, and self-esteem (H1–H3)

Table 3 reports the primary week-12 (T3) baseline-adjusted contrasts for H1–H3 (see the Introduction section, where the designation of these as primary, non-pre-registered hypotheses is discussed). All three outcomes showed a significantly higher week-12 score in the aerobics condition than the comparison condition, adjusting for the corresponding baseline score, sex, age, BMI, baseline physical activity, and prior aerobics experience, with cluster-robust standard errors: body awareness, *b* = 0.43, 95% CI [0.29, 0.56], outcome-standardized *β* = 0.51; body appreciation, *b* = 0.18, 95% CI [0.09, 0.28], outcome-standardized *β* = 0.25; and self-esteem, *b* = 0.77, 95% CI [0.62, 0.91], outcome-standardized *β* = 0.14. All three between-condition contrasts were corroborated by the wild cluster bootstrap-t procedure (all 256 Rademacher sign combinations enumerated for the eight classes, with each replicate’s own cluster-robust *t*-statistic compared to the observed *t*-statistic): all three outcomes attained the smallest two-sided *p*-value attainable under the exhaustive 256-combination sign-flip procedure (2/256 ≈ 0.0078). After Holm’s sequential correction across the three primary outcomes, all three remained significant (Holm-adjusted *p* = 0.023 for each). Hedges’ *g*, with its 95% confidence interval estimated by class-cluster bootstrap rather than by the conventional independent-samples formula, was 0.78 [0.54, 1.06] for body awareness, 0.55 [0.31, 0.81] for body appreciation, and 0.44 [0.36, 0.53] for self-esteem. Raw change-score sensitivity analyses (not covariate-adjusted for T0 as a separate regressor) produced substantively similar conclusions. Together, these class-cluster-aware analyses were consistent with H1–H3 at the primary week-12 time point.

Secondary, descriptive trajectory analyses using the same baseline-adjusted, cluster-robust specification (without the wild bootstrap or Holm correction, and not treated as additional primary tests) showed a broadly similar pattern at weeks 4 and 8 (Figure 2). Body awareness differed significantly at week 4 [*b* = 0.24, 95% CI (0.15, 0.32)] and week 8 [*b* = 0.29, 95% CI (0.17, 0.40)]. Body appreciation differed significantly at week 4 [*b* = 0.13, 95% CI (0.04, 0.23)] and week 8 [*b* = 0.18, 95% CI (0.08, 0.28)]. The week-4 self-esteem contrast was positive but imprecisely estimated and its 95% CI included zero [*b* = 0.22, 95% CI (−0.06, 0.49)]; the week-8 contrast was larger and its interval excluded zero [*b* = 0.59, 95% CI (0.24, 0.94)]. Given the small number of classes, do not interpret the week-4 result as evidence of no between-condition difference. An omnibus likelihood-ratio test (comparing, by maximum-likelihood estimation, a mixed model with a condition-by-time interaction against one without it, ignoring class-level clustering and covariates) was consistent with the two conditions’ trajectories differing overall for each outcome [body awareness, *χ*^2^(3) = 30.13, *p* < 0.001; body appreciation, *χ*^2^(3) = 16.80, *p* = 0.001; self-esteem, *χ*^2^(3) = 13.00, *p* = 0.005]; this omnibus, unadjusted model is reported only as descriptive context (full coefficients in Supplementary Table S1) and is not a primary or cluster-aware inferential test (see Table 2).

**Figure 2:**
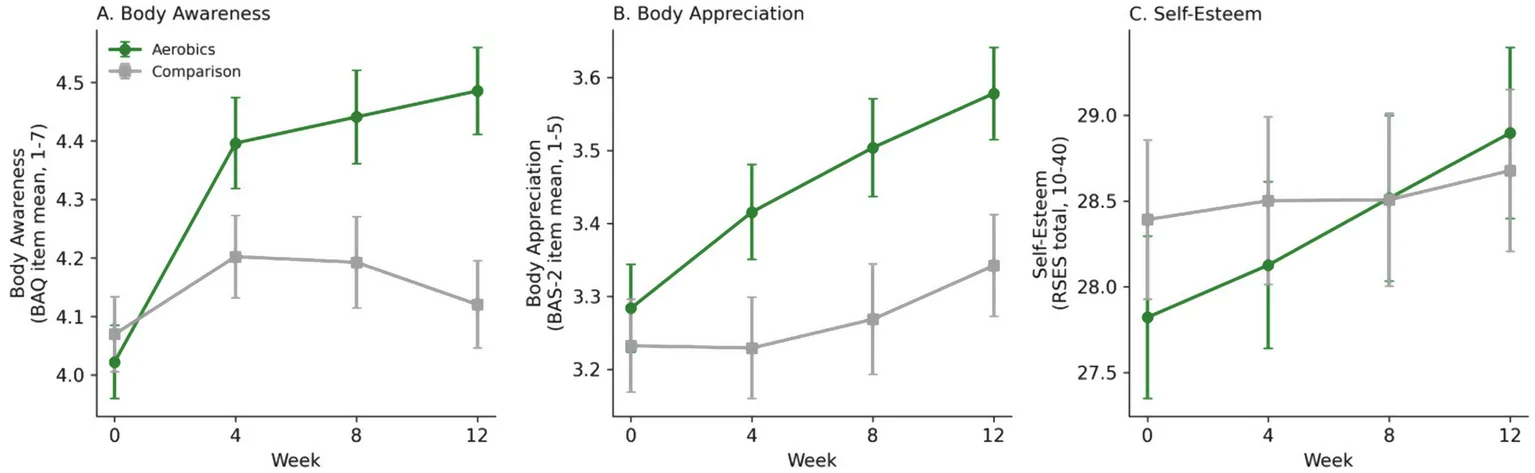
Raw (unadjusted) means with 95% confidence intervals for **(A)** body awareness, **(B)** body appreciation, and **(C)** self-esteem across the 12-week course, by condition. Aerobics, *n* = 120; comparison, *n* = 120 at each assessment wave. Colors are colorblind-safe (green/gray), and groups are additionally distinguished by marker shape (circle vs. square).

**Table 2:** Raw (unadjusted) means and SDs by condition and assessment wave.

Outcome (theoretical range)	Aerobics T0	T1	T2	T3	Comp. T0	T1	T2	T3
Body awareness (1–7)	4.02 (0.69)	4.40 (0.85)	4.44 (0.88)	4.49 (0.81)	4.07 (0.70)	4.20 (0.77)	4.19 (0.85)	4.12 (0.82)
Body appreciation (1–5)	3.28 (0.66)	3.42 (0.71)	3.50 (0.74)	3.58 (0.69)	3.23 (0.70)	3.23 (0.76)	3.27 (0.83)	3.34 (0.77)
Self-esteem (10–40)	27.82 (5.19)	28.13 (5.32)	28.52 (5.28)	28.90 (5.47)	28.39 (5.09)	28.50 (5.35)	28.51 (5.53)	28.68 (5.17)

T0 = baseline (week 0); T1 = week 4; T2 = week 8; T3 = week 12. Comp., comparison physical-education condition.

**Table 3:** Primary and secondary between-condition contrasts.

Outcome	Time	Adj. diff. (*b*)	Outcome-standardized *β*	Cluster-robust 95% CI	Enumerated wild-bootstrap p	Holm-adjusted *p*	Hedges’ *g* [cluster CI]
Body awareness	Week 12 (primary)	0.43	0.51	[0.29, 0.56]	0.0078	0.023	0.78 [0.54, 1.06]
Body appreciation	Week 12 (primary)	0.18	0.25	[0.09, 0.28]	0.0078	0.023	0.55 [0.31, 0.81]
Self-esteem	Week 12 (primary)	0.77	0.14	[0.62, 0.91]	0.0078	0.023	0.44 [0.36, 0.53]
Body awareness	Week 4 (secondary)	0.24	—	[0.15, 0.32]	—	—	—
Body awareness	Week 8 (secondary)	0.29	—	[0.17, 0.40]	—	—	—
Body appreciation	Week 4 (secondary)	0.13	—	[0.04, 0.23]	—	—	—
Body appreciation	Week 8 (secondary)	0.18	—	[0.08, 0.28]	—	—	—
Self-esteem	Week 4 (secondary)	0.22	—	[−0.06, 0.49]	—	—	—
Self-esteem	Week 8 (secondary)	0.59	—	[0.24, 0.94]	—	—	—

All rows use a baseline-adjusted ANCOVA (outcome ~ condition + own T0 score + sex + age + BMI + baseline physical activity + prior aerobics experience) with cluster-robust (CR1) standard errors. The week-12 rows are the designated primary analysis for H1–H3 (see Introduction section); exact wild-bootstrap *p*-values (all 256 Rademacher sign combinations for *k* = 8 classes) and Holm-adjusted *p*-values across the three primary outcomes are reported only for these rows. Week-4 and week-8 rows are secondary, descriptive trajectory analyses (cluster-robust CI only; no wild bootstrap or Holm correction applied). Hedges’ *g* is a descriptive standardized effect size from individual change scores; its 95% CI was estimated by class-cluster bootstrap. Supplementary Table S1 reports an unadjusted, non-cluster-robust mixed-effects model underlying the omnibus likelihood-ratio tests for change in body awareness, body appreciation, and self-esteem.

### Class-level sensitivity analysis

As a further check using the class rather than the individual as the unit of analysis, mean T0-to-T3 change was computed separately for each of the eight classes (Supplementary Table S3). All four aerobics classes showed larger mean change than all four comparison classes on every outcome, with no overlap between the two sets of four class means. Comparing the four aerobics-class means against the four comparison-class means (*n* = 4 vs. 4, a test with very limited power) gave the following: body awareness, class-level *t*(6) = 5.26, *p* = 0.002, exact Mann–Whitney *p* = 0.029; body appreciation, *t*(6) = 3.53, *p* = 0.012, exact Mann–Whitney *p* = 0.029; self-esteem, *t*(6) = 9.84, *p* < 0.001, exact Mann–Whitney *p* = 0.029. This class-level pattern was directionally consistent with the individual-level, cluster-robust results and is presented as a sensitivity check rather than as strong independent corroboration. However, with only four classes per condition, this analysis has limited ability to distinguish condition differences from class-specific or instructor-specific influences.

### Associations among body awareness, body appreciation, and self-esteem (H4–H5)

Consistent with H4, week-4 body awareness was positively associated with week-8 body appreciation in a cluster-robust regression controlling for baseline body awareness, baseline body appreciation, and condition [*b* = 0.36, class-cluster-robust 95% CI (0.25, 0.47), standardized *β* = 0.37, *p* < 0.001]; condition itself remained associated with week-8 body appreciation in this model (*b* = 0.09, *p* = 0.027), consistent with the direct pathway reported for H6 (see Exploratory Mediation Analyses section, below). The corresponding zero-order correlation, which treats participants as independent and does not control for condition, is reported only for descriptive comparison: *r*(238) = 0.577, *p* < 0.001. Consistent with H5, week-8 body appreciation was positively associated with week-12 self-esteem in an analogous cluster-robust regression controlling for baseline body appreciation, baseline self-esteem, and condition [*b* = 2.07, 95% CI (1.59, 2.56), standardized *β* = 0.31, *p* < 0.001]; condition also remained associated with week-12 self-esteem in this model (*b* = 0.43, *p* = 0.004). Zero-order *r*(238) = 0.652, *p* < 0.001. Because both focal constructs are themselves associated with condition (see change in body awareness, body appreciation, and self-esteem section, above), controlling for condition in these models reduces the risk that the body-awareness–body-appreciation and body-appreciation–self-esteem associations simply reflect shared intervention exposure; these models did not include the fuller covariate set (sex, age, BMI, physical activity, prior experience) used in the primary intervention models (see Statistical Analysis section, above).

### Exploratory Mediation Analyses (H6–H7)

H6 (exploratory). In a two-path, class-cluster-bootstrapped model with Δ body appreciation as the terminal outcome, the indirect path from condition through Δ body awareness to Δ body appreciation was different from zero [*a*₁*d*₂₁ = 0.087, 95% CI (0.051, 0.137)]. This pattern is consistent with H6; however, as an exploratory, non-pre-registered analysis of observational (non-randomized) data, it does not establish that body awareness causally precedes body appreciation.

H7 (exploratory). In the class-cluster-bootstrapped serial mediation model illustrated in Figure 3, an indirect estimate following the hypothesized construct ordering—condition, then Δ body awareness, then Δ body appreciation, then Δ self-esteem—was different from zero [*a*₁*d*₂₁*b*₂ = 0.146, 95% CI (0.079, 0.240)]. Because that ordering was imposed by the analyst to match the hypothesized sequence rather than derived independently from the timing of measurement (see Study Design section, above), this result is a statistical association consistent with the hypothesized chain, not a demonstration that body awareness changes first and causes subsequent change in body appreciation. This estimate was also similar in magnitude to two parallel estimates from the same model—through body awareness alone and through body appreciation alone—so the data do not indicate that the specific serial ordering is a stronger mechanism than either construct operating independently; the full set of path coefficients, both mediators’ independent pathways, and a supplementary participant-level bootstrap are reported in Supplementary Table S2. rather than in the main text, because condition varied at the class level and a participant-level resampling scheme does not match how condition was assigned. Given the quasi-experimental design, the small number of classes, and the overlapping change intervals used to order the mediators (see Study Design section, above), this serial indirect estimate should be read as descriptive and hypothesis-generating rather than as confirmation of the proposed mechanism, and we do not report a specific percentage of the total effect “explained” by the two mediators given the instability such ratios can show with only eight clusters.

**Figure 3:**
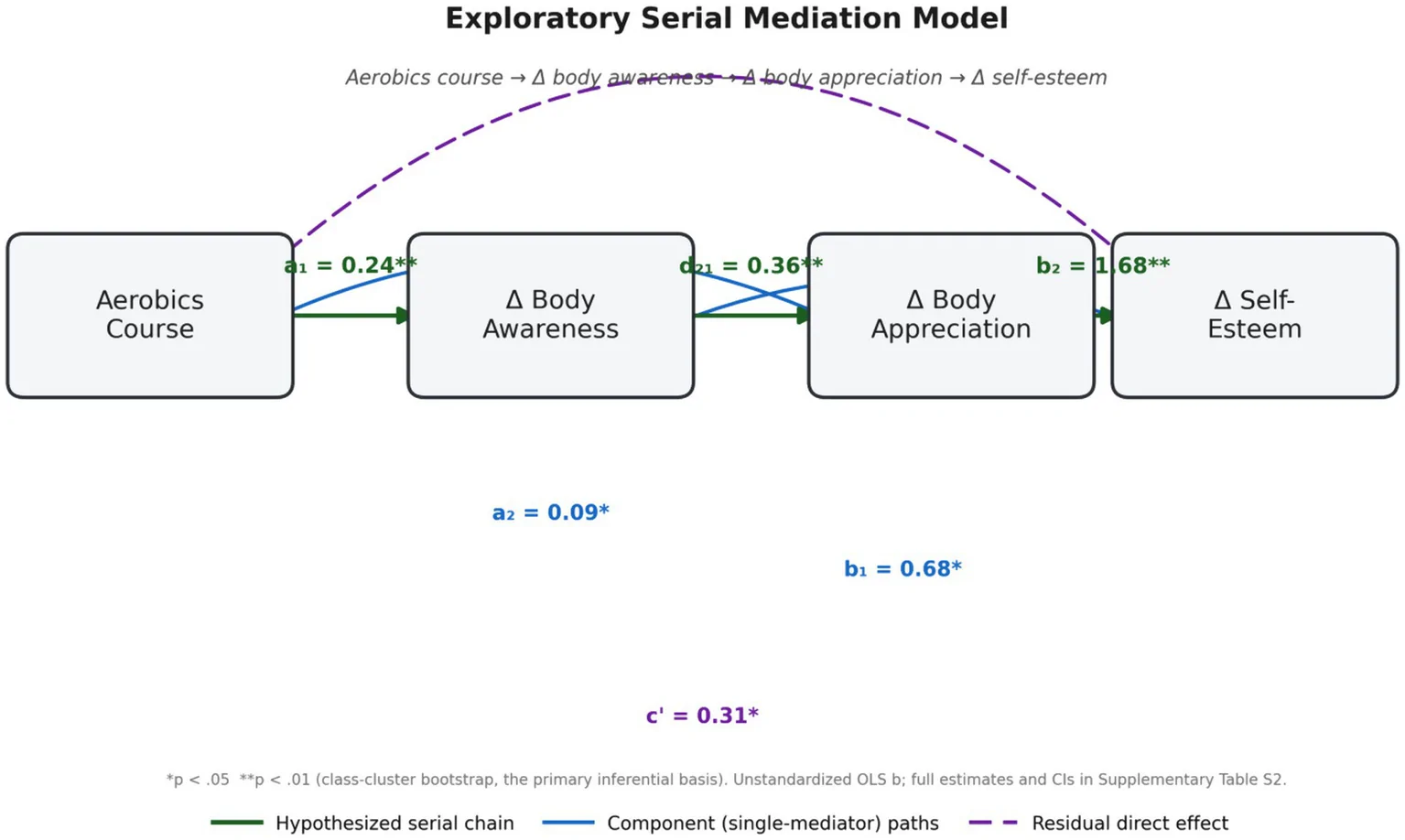
Exploratory serial mediation model: course condition (aerobics vs. comparison) → Δ body awareness → Δ body appreciation → Δ self-esteem. The top row shows the hypothesized serial chain (green); nested arcs below show the two component (single-mediator) paths (blue) and the residual direct effect (purple, dashed). All six paths shown are different from zero under the primary class-cluster bootstrap (Supplementary Table S2); the residual direct effect was not significant only under the supplementary participant-level bootstrap, which does not match how the condition was assigned (see Exploratory Mediation Analyses section, above). Coefficients are unstandardized OLS *b*, class-cluster-bootstrapped; full estimates and confidence intervals appear in Supplementary Table S2. The arrows represent modeled statistical paths and should not be interpreted as demonstrating causal effects or an aerobics-specific mechanism.

## Discussion

Students enrolled in the aerobics classes showed more favorable changes than students in the incompletely characterized comparison classes on all three outcomes at the primary week-12 contrast: body awareness (*g* = 0.78), body appreciation (*g* = 0.55), and self-esteem (*g* = 0.44). In standardized terms, the between-condition difference was largest for body awareness, moderate for body appreciation, and comparatively smaller for self-esteem. This pattern suggests that the clearest practical signal of the course was in students’ self-reported attentiveness to bodily states, whereas changes in broader body appreciation and especially global self-esteem were more modest. These effect sizes should nevertheless be interpreted cautiously because they describe between-course differences rather than isolated causal effects of aerobics. These between-condition findings should be interpreted at the class level: condition was defined by eight intact classes, with only four classes contributing independent replication within each condition. Thus, although 240 students contributed repeated outcome data, the precision and causal interpretability of the between-condition contrasts are constrained primarily by the small number of classes rather than by the number of individual observations. The between-condition difference in body awareness reached significance earlier in the secondary trajectory analyses (week 4) than the difference in self-esteem (week 8); body appreciation followed a similar early pattern that then leveled off after week 8 rather than continuing to grow. Although this descriptive timing is compatible with the hypothesized ordering, it should not be interpreted as evidence of temporal precedence because the underlying change intervals overlap and the analytic model specified the mediator ordering *a priori* rather than establishing it independently through the measurement schedule. The exploratory mediation analysis produced an indirect estimate corresponding to the hypothesized ordering—condition, then change in body awareness, then change in body appreciation, then change in self-esteem—that was different from zero. However, this finding does not provide evidence that the sequential model is superior to alternative models. The serial indirect estimate (0.146) was similar in magnitude to the indirect pathway through body awareness alone (0.162) and the pathway through body appreciation alone (0.157; Supplementary Table S2). Thus, the present data are compatible with a sequential awareness-to-appreciation pathway, but also with parallel contributions of body awareness and body appreciation, and with alternative temporal or reciprocal relations among these constructs. The mediation findings therefore should not be interpreted as establishing a privileged or dominant psychological mechanism.

These results are compatible with embodiment-based accounts of body-attentive movement (Daubenmier, 2005; Piran, 2017) and with functionality-based models of positive body image (Alleva and Tylka, 2021; Tylka and Wood-Barcalow, 2015a), which propose that shifting the basis of self-evaluation toward internal bodily experience is associated with more favorable body appreciation and, in turn, broader self-worth. These interpretations should also be understood as context-specific, because the study involved young undergraduates participating in existing physical-education courses within a single Chinese university. This theoretical compatibility should not be taken as evidence that the embodiment-based sequential account is uniquely supported by the present data. They are also consistent with recent evidence linking exercise-related constructs to body appreciation and self-esteem in similar populations (Shi et al., 2024). This compatibility should not be interpreted as evidence that aerobics-specific features drove the observed between-course differences. However, several alternative, non-body-image explanations for the observed condition difference cannot be ruled out by this design: differences in instructor style or enthusiasm between the aerobics and comparison sections, the inherent enjoyment or novelty of aerobics choreography relative to standard physical education, heightened social interaction or group cohesion fostered by synchronized group movement and shared music, differences in objective exercise intensity between conditions, self-selection of more body-motivated or change-ready students into the aerobics elective, and participants’ awareness of the study’s likely hypotheses given repeated body-focused questionnaires alongside a body-focused course. The present data therefore cannot adjudicate among competing psychological models involving sequential, parallel, or alternative temporal relations among body awareness, body appreciation, and self-esteem. Because these characteristics may vary systematically at the class level, increasing the number of students within the same eight classes cannot resolve this uncertainty; replication across more classes and instructors would be required.

From an applied perspective, the present mediation findings do not indicate whether body awareness, body appreciation, or their sequential ordering should be preferentially targeted in university physical-education programs. The three indirect pathways were of broadly similar magnitude, and the mediators were not experimentally manipulated. Accordingly, the present study cannot determine whether an awareness-then-appreciation sequence would be more effective than targeting either construct independently or simultaneously. Experimental studies that manipulate these components separately would be required to distinguish among these possibilities.

## Limitations

Group assignment was not randomized: students self-selected into intact class sections before the study began, so despite reasonable baseline equivalence (Table 1), unmeasured selection factors cannot be ruled out as alternative explanations for the observed differences. The condition was implemented across only eight intact classes (four per condition). Accordingly, the class rather than the individual student was the effective unit of condition assignment and independent replication for between-condition inference. Although cluster-robust standard errors, wild-cluster bootstrap inference based on exhaustive sign enumeration, and class-level sensitivity analyses were used, the small number of independent classes limits statistical precision and does not eliminate class-level confounding.

The comparison condition was an incompletely characterized usual-practice physical-education condition rather than a standardized active control. Information on its specific activities, instructors, use of music or synchronized movement, attendance procedures, exercise intensity, and implementation fidelity was unavailable. Consequently, the observed between-condition differences cannot be attributed specifically to aerobics choreography or body-focused movement and may reflect other instructional or contextual differences between courses. In addition, intervention attendance could not be reliably verified because the original attendance registers and the operational definition of the available sessions-attended variable were not retained; therefore, individual intervention exposure could not be quantified reliably.

The absence of an *a priori* power analysis is an additional limitation. Given the small number of independent classes, modest between-condition effects may be difficult to detect; therefore, interpret non-significant secondary contrasts, particularly the week-4 self-esteem estimate, as inconclusive rather than evidence of no difference.

The exploratory mediation model also does not establish causal or strict temporal precedence. Because the change intervals overlap, the findings are consistent with, but do not confirm, the hypothesized sequential mechanism. Alternative temporal orderings, parallel pathways, and reciprocal relations remain plausible.

All measures were self-report. In addition, only scale-level composite scores were retained in the de-identified analytic dataset; therefore, item-level scoring cannot now be independently reconstructed, sample-specific reliability coefficients cannot be calculated, and measurement invariance cannot be examined. These measurement limitations should be considered when interpreting the findings.

Generalizability is limited because participants were undergraduate students aged 18–24 from a single university in China. Replication across additional institutions and populations is needed. Finally, exact original class enrollment, numbers excluded before sampling, and counts declining or replaced after sampling were not fully recoverable from archived records, although the available records confirmed the fixed sampling of 30 participants per class and complete four-wave data for the final analytic sample (see Figure 1).

## Conclusion

Students enrolled in the aerobics classes had more favorable changes in body awareness, body appreciation, and self-esteem than students in the comparison physical-education classes. The magnitude of these differences was greatest for body awareness, intermediate for body appreciation, and smaller for self-esteem. Because the comparison curriculum was not fully documented and course enrollment was non-random, the findings should be interpreted as a preliminary between-course association rather than as evidence of an aerobics-specific causal effect. Exploratory Mediation Analyses were consistent with statistical associations involving body awareness and body appreciation, but did not distinguish a sequential awareness-to-appreciation mechanism from alternative or parallel pathways. Replication with a substantially larger number of independently observed classes and instructors, ideally within randomized, multi-site designs with fully documented intervention and comparison protocols, is needed before practical recommendations can be made.

## Data Availability

The original contributions presented in the study are included in the article/Supplementary material, further inquiries can be directed to the corresponding authors.
