# Supplementary material for: Changes in body awareness, body appreciation, and self-esteem during a university aerobics course: a quasi-experimental study
Source: Front Psychol. 2026 Oct 19;17:1952535. doi: 10.3389/fpsyg.2026.1952535 (PMC13724576; doi:10.3389/fpsyg.2026.1952535)
Supplement: Supplementary file 1 [file Table_2.DOCX]

**Supplementary Table S3. Class-level mean T0-to-T3 change for each of the eight classes. BAQ = body awareness; BAS2 = body appreciation; RSES = self-esteem.**

| **Class** | **n** | **BAQ change** | **BAS2 change** | **RSES change** | **Condition** |
| --- | --- | --- | --- | --- | --- |
| C01 | 30 | 0.509 | 0.340 | 1.236 | Aerobics |
| C03 | 30 | 0.318 | 0.222 | 1.139 | Aerobics |
| C06 | 30 | 0.378 | 0.418 | 0.930 | Aerobics |
| C08 | 30 | 0.646 | 0.199 | 0.994 | Aerobics |
| C02 | 30 | 0.015 | 0.104 | 0.254 | Comparison |
| C04 | 30 | 0.044 | 0.106 | 0.218 | Comparison |
| C05 | 30 | 0.010 | 0.137 | 0.268 | Comparison |
| C07 | 30 | 0.134 | 0.092 | 0.403 | Comparison |
